# Long-range repression by ecdysone receptor on complex enhancers of the insulin receptor gene

**DOI:** 10.1080/19336934.2023.2242238

**Published:** 2023-08-24

**Authors:** Katie D. Thompson, Will Suber, Rachel Nicholas, David N. Arnosti

**Affiliations:** a Department of Biochemistry and Molecular Biology, Michigan State University, East Lansing, MI, USA; b Department of Microbiology and Molecular Genetics, Michigan State University, East Lansing, MI, USA

**Keywords:** Insulin, insulin receptor, Drosophila, enhancer, dFOXO, ecdysone, 20E, ecdysone receptor

## Abstract

The insulin signalling pathway is evolutionarily conserved throughout metazoans, playing key roles in development, growth, and metabolism. Misregulation of this pathway is associated with a multitude of disease states including diabetes, cancer, and neurodegeneration. The human insulin receptor gene (*INSR)* is widely expressed throughout development and was previously described as a ‘housekeeping’ gene. Yet, there is abundant evidence that this gene is expressed in a cell-type specific manner, with dynamic regulation in response to environmental signals. The Drosophila insulin-like receptor gene (*InR*) is homologous to the human *INSR* gene and was previously shown to be regulated by multiple transcriptional elements located primarily within the introns of the gene. These elements were roughly defined in ~1.5 kbp segments, but we lack an understanding of the potential detailed mechanisms of their regulation. We characterized the substructure of these cis-regulatory elements in Drosophila S2 cells, focusing on regulation through the ecdysone receptor (EcR) and the dFOXO transcription factor. By identifying specific locations of activators and repressors within 300 bp subelements, we show that some previously identified enhancers consist of relatively compact clusters of activators, while others have a distributed architecture not amenable to further reduction. In addition, these assays uncovered a long-range repressive action of unliganded EcR. The complex transcriptional circuitry likely endows *InR* with a highly flexible and tissue-specific response to tune insulin signalling. Further studies will provide insights to demonstrate the impact of natural variation in this gene’s regulation, applicable to human genetic studies.

## Introduction

The conserved insulin signalling pathway plays a key role in development, reproduction, growth and metabolism in metazoans [1–4]. In mammals, insulin is released by the pancreas and binds to the insulin receptor (IR), a receptor kinase receptor that can be autophosphorylated, activating various metabolic pathways including the phosphatidylinositol 3-kinase (PI3K/AKT) pathway. Stimulation of this pathway is important for metabolic activity and furthermore involves the Ras-mitogen-activated protein kinase (MAPK), which is responsible for cell growth and development [5,6]. FOXO is a key transcription factor that is phosphorylated in response to activation of the insulin signalling pathway. When FOXO is phosphorylated, it is excluded from the nucleus, abrogating the positive activity of this transcription factor on the insulin receptor gene (*INSR*), thus the FOXO-*INSR* relationship represents a negative feedback loop that may fine-tune levels of signalling [7,8]. Additional studies show further complexities to FOXO regulation; it has been shown that kinase signalling can inactivate FOXO even when prevented from redistribution to the cytoplasm, likely based on inhibition of DNA binding [8,9].

Signalling through the insulin receptor has been associated with a number of conditions relevant to human health. Rare mutations in the insulin receptor protein coding sequence can give rise to severe growth defects and insulin resistance [10,11], while changes in the expression and regulation of *INSR* can affect overall signalling and has been associated with diabetes, cancer, and neurodegenerative diseases [12–16]. In type II diabetes, insulin binds to the insulin receptor, but there is a failure to activate the insulin signalling cascade, resulting in a loss of glucose transport into cells and ultimately high blood glucose levels [13,16]. Insulin receptors are also recycled in the cell through endocytosis as a posttranscriptional process to regulate insulin signalling, impacting insulin resistance and type II diabetes [17]. The insulin receptor gene comprises 22 exons and 21 introns; alternative splicing at exon 11 results in two isoforms of the protein, IR-B, and IR-A. IR-B is the dominant, mature isoform, while IR-A is found predominantly in fetal cells and cancer cells. The overexpression of IR-A increases the isoform ratio IR-A/IR-B up to 20-fold in some cancers and allows cancer cells to respond to insulin and insulin-like growth factors [13,15,18,19]. The insulin signalling pathway is also critical in the process of ageing and Alzheimer’s disease (AD); insulin regulates brain glucose metabolism in the brain, but in AD, there is often reduced IR expression and tyrosine kinase signalling, resulting in defects in neuronal activity and cognitive function [12,14]. The insulin receptor thus provides a potential target for therapies for these diseases, including interventions that may impact binding affinity of insulin to the receptor, by increasing expression cell surface receptors or by prevention of ubiquitination and degradation of the receptors [20]. We lack a general understanding of how *INSR* expression levels impact physiology and disease and its potential as a target for therapeutic purposes. *INSR* has been previously described as a ‘housekeeping’ gene because of its broad expression; however, there is evidence of cell-type specificity and cis-regulatory elements [21–23].

The human *INSR* and the *InR* in Drosophila are homologous genes that are broadly expressed. The genes include large introns that have been demonstrated to contain chromatin features predicted to possess enhancer function or have been demonstrated to direct transcription [22,23]. In insects, the insulin signalling pathway has also been found in most tissues and shown to be crucial for embryonic development, neuronal function, and organ growth [1,3,24]. Insulin-like peptides, DILPs, are expressed by insulin-producing cells in the gut, fat body, and brain; these peptides bind to the insulin-like receptor, InR, leading to a similar insulin signalling cascade as seen in mammals [1,25]. In Drosophila, as in mammals, the insulin signalling pathway responds to the environment and signalling inputs to regulate gene expression of the insulin receptor. Signalling involves the action of dFOXO, the Drosophila homolog of FOXO [7,8].

A central steroid hormone in Drosophila is 20-hydroxyecdysone (20E), which binds to the ecdysone receptor (EcR) and triggers activation of diverse genes that are critical for development. Surges of ecdysone during different developmental stages engage batteries of genes that can be distinguished between early/late genes [26–28]. EcR has been shown to directly bind throughout the entire Drosophila genome, usually within 10 kbp of 20E-regulated genes in a tissue-specific manner. The pervasive effects of ecdysone signalling can be attributed to the many transcription factors whose genes are targeted by this pathway [29]. Ecdysone has been shown to regulate *InR* expression, but there is a lack of understanding of the direct action of EcR on the *InR* locus [29,30].

**Figure 1.**
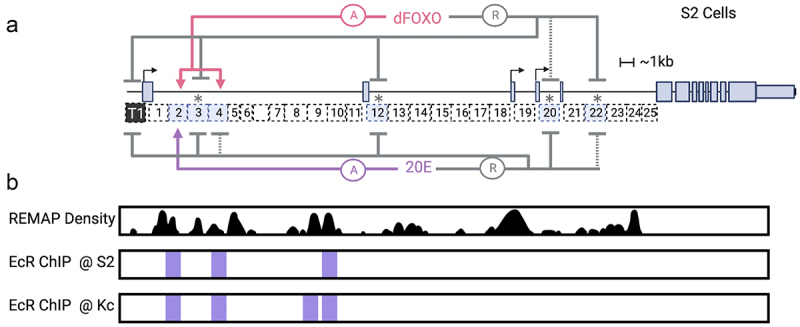
Regulatory elements of the Drosophila *InR* locus in S2 cell culture. a) a diagram illustrating previously identified enhancers of the *InR* locus [23] the enhancers marked with an asterisk represent elements with intrinsically substantial activity, independent of dFOXO overexpression or 20E treatment. b) REMAP 2022 Density showing predicted regulatory regions throughout the *InR* locus [31]. REMAP is a large-scale integrative analysis of transcriptional regulators that catalogues the results of ChIP-seq, ChIP-exo, and DAP-seq. EcR ChIP-seq peaks in S2 and Kc cells from the UCSC Genome Browser (Recreated with Adobe Illustrator).

We previously identified discrete regulatory regions within the intronic regions of the *InR* locus in Drosophila. Utilizing luciferase assays in cultured *Drosophila melanogaster* S2 and Kc cells, the ~40 kbp of intronic regions were assessed as 25 subfragments of ~1.5 kbp each (1–25) including those responsive to dFOXO overexpression or 20E treatment (Figure 1). The active elements were then further mutagenized by specific serial deletions to identify parts of the enhancer that were necessary for activity. Some enhancers functioned in a cell-type-specific manner, with preferential action in S2 vs. Kc cells [23].

In this study, we sought to learn about the molecular organization of these putative *InR* enhancer elements by testing for sufficiency of small regulatory blocks and not merely necessity, as in our earlier work. Our analysis of sub-elements 300 bp and 600 bp in size revealed diverse activities; in some cases, the action of the larger regulatory region can be summarized as the activity of one or two individual small elements, but in other cases, activity was dependent on much larger fragments, suggesting the cooperative action of factors binding across these elements. Particularly striking was the identification of a long-range repressor action mediated by the ecdysone receptor, a protein that has largely been studied in the context of promoter-proximal activity [32].

## Materials & methods

### Cloning

The *InR* introns were previously divided into ~1.5 kbp fragments; the fragments that showed intrinsic activity and/or response to dFOXO or 20E were divided further into ~300 bp and ~600 bp (a-e and ab-de). Enhancer 22, which is longer than 1.5kb, was divided into (22a-f, and 22ab-ef). The 300 bp and 600 bp fragments were cloned into the same luciferase reporter vector as previously described by [23]. The vector, p2T-Luc, is a luciferase reporter vector that also contains an ampicillin-resistant gene, and the intronic fragments were cloned upstream of the T1 promoter between the Not1 and Asc1 sites of the vector as previously described [23]. We utilized the transcription factor dFOXO previously cloned into the pAX vector [23]. All primers can be found in the table (Table 1) and were ordered from Integrated DNA Technologies, IDT [33]. All cloned fragments were checked for accuracy through Sanger sequencing at Michigan State University Research Technology Support Facility.

**Table 1.** List of DNA oligonucleotides, shown 5' to 3', used to produce tested regulatory elements.

	PCR Primers Name		PCR Primer Sequence (5’ − 3’)		
Fragment Name	Not1	AscI	Not1	Asc1	Fragment Length (bp)
2	DA 6741	DA 6750	TATTAAGCGGCCGCATCGCTTCTTGGAACAATC	TCTTAAGGCGCGCCAACAGGCAAAACCGAAGTCAG	1505
2a	DA 6749	DA 6750	TCTTAAGCGGCCGCCTCTATAGATCTCTTCTGTTCTTC	TCTTAAGGCGCGCCAACAGGCAAAACCGAAGTCAG	302
2b	DA 6747	DA 6748	TCTTAAGCGGCCGCTAGACATGTGGTCAGAGC	TATTAAGGCGCGCCGAGGCAATGACGGAACCCGG	312
2c	DA 6745	DA 6746	TATTAAGCGGCCGCAGCAGTGAAGGCAGCAGC	TCTTAAGGCGCGCCCCACATGTCTAAACGTAGCTG	306
2d	DA 6743	DA 6744	TCTTAAGCGGCCGCGTCGCTTAAATAAATTAGTAG	TATTAAGGCGCGCCTGCTGCTTATGCTGTGAACTGCTTG	303
2e	DA 6741	DA 6742	TATTAAGCGGCCGCATCGCTTCTTGGAACAATC	TCTTTAAGGCGCGCCCTCAAATACAAGTTACAAATATG	300
**2a-b**	DA 6747	DA 6750	TCTTAAGCGGCCGCTAGACATGTGGTCAGAGC	TCTTAAGGCGCGCCAACAGGCAAAACCGAAGTCAG	614
**2b-c**	DA 6745	DA 6748	TATTAAGCGGCCGCAGCAGTGAAGGCAGCAGC	TATTAAGGCGCGCCGAGGCAATGACGGAACCCGG	618
**2c-d**	DA 6743	DA 6746	TCTTAAGCGGCCGCGTCGCTTAAATAAATTAGTAG	TATTAAGGCGCGCCGAGGCAATGACGGAACCCGG	609
2d-e	DA 6741	DA 6744	TATTAAGCGGCCGCATCGCTTCTTGGAACAATC	TATTAAGGCGCGCCTGCTGCTTATGCTGTGAACTGCTTG	603
3	DA 6731	DA 6740	TCTTAAGCGGCCGCCAGTGAAAAATAGGCAACAAC	TATTAAGGCGCGCCGTTCCAAGAAGCGATGCGAGGGC	1743
3a	DA 6739	DA 6740	TATTAAGCGGCCGCTACGACCGGCTGACGAAC	TATTAAGGCGCGCCGTTCCAAGAAGCGATGCGAGGGC	348
3b	DA 6737	DA 6738	TATTAAGCGGCCGCGTGAAAACCATAATTGTACACAC	TATTAAGGCGCGCCGACGAGAGTCTCCCCCGA	351
3c	DA 6735	DA 6736	TATTAAGCGGCCGCTGCCATTGGCCAATTGTTG	TCTTAAGGCGCGCCCACTATGTGATTTAAAGAACAGC	349
3d	DA 6733	DA 6734	TATTAAGCGGCCGCCTGCAAAGGCGGAGATAAC	TATTAAGGCGCGCCTGTTTGTTTTGATATTTTGATTCC	346
3e	DA 6731	DA 6732	TCTTAAGCGGCCGCCAGTGAAAAATAGGCAACAAC	TCTTAAGGCGCGCCGTAGAATTAAAATTGTTATTTTG	347
3a-b	DA 3737	DA 6740	TATTAAGCGGCCGCGTGAAAACCATAATTGTACACAC	TATTAAGGCGCGCCGTTCCAAGAAGCGATGCGAGGGC	700
3b-c	DA 6735	DA 6738	TATTAAGCGGCCGCTGCCATTGGCCAATTGTTG	TATTAAGGCGCGCCGACGAGAGTCTCCCCCGA	697
3c-d	DA 6733	DA 6736	TATTAAGCGGCCGCCTGCAAAGGCGGAGATAAC	TCTTAAGGCGCGCCCACTATGTGATTTAAAGAACAGC	698
3d-e	DA 6731	DA 6734	TCTTAAGCGGCCGCCAGTGAAAAATAGGCAACAAC	TATTAAGGCGCGCCTGTTTGTTTTGATATTTTGATTCC	693
4	DA 5334	DA 5335	AATTTAAGCGGCCGCTGTGTTGTTGCCTATTTTTCACTGT	AATTAAGGCGCGCCAGGTGACAACGTGCGAGATT	1576
4a	DA 7368	DA 7369	AATTAAGGCGCGCCTGTGTTGTTGCCTATTTTTCAC	AATTAAGCGGCCGCACGAAAACGGAATCTTTC	315
4b	DA 7370	DA 7371	AATTAAGGCGCGCCATTGACATTCGTTTATTATTTTCCG	AATTAAGCGGCCGCTATCAAAACCGAAGCGCAGCC	315
4c	DA 7372	DA 7373	AATTAAGGCGCGCCCCCGTTAGATAACAATGGG	AATTAAGCGGCCGCATGACTATAAATACAATAC	315
4d	DA 7374	DA 7375	AATTAAGGCGCGCCTTTCATAAACATTACGAGATAAGC	AATTAAGCGGCCGCCGGGCGAAAGAACGAACTAAGGC	315
4e	DA 7376	DA 7377	AATTAAGGCGCGCCTGAATGACCTTCGCCTGTCCCGC	AATTAAGCGGCCGCAGGTGACAACGTGCGAGATTAAAG	316
4a-b	DA 7368	DA 7371	AATTAAGGCGCGCCTGTGTTGTTGCCTATTTTTCAC	AATTAAGCGGCCGCTATCAAAACCGAAGCGCAGCC	630
4b-c	DA 7370	DA 7373	AATTAAGGCGCGCCATTGACATTCGTTTATTATTTTCCG	AATTAAGCGGCCGCATGACTATAAATACAATAC	630
4c-d	DA 7372	DA 7375	AATTAAGGCGCGCCCCCGTTAGATAACAATGGG	AATTAAGCGGCCGCCGGGCGAAAGAACGAACTAAGGC	630
4d-e	DA 7374	DA 7377	AATTAAGGCGCGCCTTTCATAAACATTACGAGATAAGC	AATTAAGCGGCCGCAGGTGACAACGTGCGAGATTAAAG	631
12	DA 6909	DA 6918	TATTAAGCGGCCGCAGCTTGGGCTTTACCTTCTTC	TATTAAGGCGCGCCTGGACTTTCGAATATGCATG	1612
12a	DA 6909	DA 6910	TATTAAGCGGCCGCAGCTTGGGCTTTACCTTCTTC	TATTAAGCCGCGCCACAGCATACATTTCTTAGG	322
12b	DA 6911	DA 6912	TATTAAGCGGCGCTTTGATTGTCAATGGGGAATTTG	TATTAAGGCGCGCCGACGCGCTATTTGTTACAC	322
12c	DA 6913	DA 6914	TATTAAGCGGCCGCTGTCGCAACAAACAACTGC	TATTAAGGCGCGCCCCACAGTGCCATAGGAACG	323
12d	DA 6915	DA 6916	TATTAAGCGGCCGCCCATATAGCTTCTTACATATATG	TATTAAGGCGCGCCCATGGTGCCACACACTAATAG	322
12e	DA 6917	DA 6918	TATTAAGCGGCCGCTGGCATGCGGTATGCCAG	TATTAAGGCGCGCCTGGACTTTCGAATATGCATG	325
12a-b	DA 6909	DA 6912	TATTAAGCGGCCGCAGCTTGGGCTTTACCTTCTTC	TATTAAGGCGCGCCGACGCGCTATTTGTTACAC	644
12b-c	DA 6911	DA 6914	TATTAAGCGGCGCTTTGATTGTCAATGGGGAATTTG	TATTAAGGCGCGCCCCACAGTGCCATAGGAACG	645
12c-d	DA 6913	DA 6916	TATTAAGCGGCCGCTGTCGCAACAAACAACTGC	TATTAAGGCGCGCCCATGGTGCCACACACTAATAG	645
12d-e	DA 6915	DA 6918	TATTAAGCGGCCGCCCATATAGCTTCTTACATATATG	TATTAAGGCGCGCCTGGACTTTCGAATATGCATG	646
20	DA 6919	DA 6928	TATTAAGCGGCCGCTGCTTTTCGCGCCTTTCATTC	TATTAAGGCGCGCCCTTTGCAAGTGCCTGCCTTTG	1738
20a	DA 6919	DA 6920	TATTAAGCGGCCGCTGCTTTTCGCGCCTTTCATTC	TATTAAGGCGCGCCCAACATTTTTGTGGTTTTGTTG	347
20b	DA 6921	DA 6922	TATTAAGCGGCCGCTATAAGCCCCAGTGGATTTTAG	TATTAAGGCGCGCCATTTTTATATCATCCACAAC	350
20c	DA 6923	DA 6924	TATTAAGCGGCCGCAGCATGAATGAGAAAACAAG	TATTAAGGCGCGCCGTTAAAGACCGTATGTGGTTG	347
20d	DA 6925	DA 6926	TATTAAGCGGCCGCCATGTTTAGTGCCTTTAC	TATTAAGGCGCGCCTTTCGAAGTCCACACCGTAC	348
20e	DA 6927	DA 6928	TATTAAGCGGCCGCCGGGGTCTCTCGTACTCGATC	TATTAAGGCGCGCCCTTTGCAAGTGCCTGCCTTTG	350
20a-b	DA 6919	DA 6922	TATTAAGCGGCCGCTGCTTTTCGCGCCTTTCATTC	TATTAAGGCGCGCCATTTTTATATCATCCACAAC	697
20b-c	DA 6921	DA 6924	TATTAAGCGGCCGCTATAAGCCCCAGTGGATTTTAG	TATTAAGGCGCGCCGTTAAAGACCGTATGTGGTTG	697
20c-d	DA 6923	DA 6926	TATTAAGCGGCCGCAGCATGAATGAGAAAACAAG	TATTAAGGCGCGCCTTTCGAAGTCCACACCGTAC	694
20d-e	DA 6925	DA 6928	TATTAAGCGGCCGCCATGTTTAGTGCCTTTAC	TATTAAGGCGCGCCCTTTGCAAGTGCCTGCCTTTG	698
22	DA 5366	DA 5367	AATTAAGCGGCCGCCTTTTCGAAGCGGATCTCCC	AATTAAGGCGCGCCGCGAATAGTGTGTTGTGGCG	
22a	DA 5366	DA 6984	AATTAAGCGGCCGCCTTTTCGAAGCGGATCTCCC	AATTAAGGCGCGCCAACCAGAAAGCCAAGTCTCAC	255
22b	DA 6982	DA 6983	AATTAAGCGGCCGCGACTTGTTCGAACGAGTGTC	AATTAAGGCGCGCCTCATGCTGTGGCTTTAAATC	289
22c	DA 6981	DA 6980	AATTAAGCGGCCGCGATTGTATGTTTTTTAATATTTC	AATTAAGGCGCGCCAAGGGTGGAAACATGCGGGC	289
22d	DA 6979	DA 6978	AATTAAGCGGCCGCCGTTTTTCAACTCCTGTAAAG	AATTAAGGCGCGCCCAATCTTATTCCGCGGTATTTC	291
22e	DA 6977	DA 6976	AATTAAGCGGCCGCGAAAATCCCAACTTGTTTGC	AATTAAGGCGCGCCGGTGTCGCCAGTCTTACCCG	290
22f	DA 6975	DA 5367	AATTAAGCGGCCGCCACAATTGAATGTTTTAATTG	AATTAAGGCGCGCCGCGAATAGTGTGTTGTGGCG	311
22a-b	DA 5366	DA 6983	AATTAAGCGGCCGCCTTTTCGAAGCGGATCTCCC	AATTAAGGCGCGCCTCATGCTGTGGCTTTAAATC	534
22b-c	DA 6982	DA 6980	AATTAAGCGGCCGCGACTTGTTCGAACGAGTGTC	AATTAAGGCGCGCCAAGGGTGGAAACATGCGGGC	577
22c-d	DA 6981	DA 6978	AATTAAGCGGCCGCGATTGTATGTTTTTTAATATTTC	AATTAAGGCGCGCCCAATCTTATTCCGCGGTATTTC	580
22d-e	DA 6979	DA 6976	AATTAAGCGGCCGCCGTTTTTCAACTCCTGTAAAG	AATTAAGGCGCGCCGGTGTCGCCAGTCTTACCCG	580
22e-f	DA 6977	DA 5367	AATTAAGCGGCCGCGAAAATCCCAACTTGTTTGC	AATTAAGGCGCGCCGCGAATAGTGTGTTGTGGCG	600

### Transfections

*Drosophila melanogaster* S2 cells obtained from the Drosophila Genomics Resource Center at the Indiana University of Bloomington were used for the cellular assays. About 1.5 mL of 1–1.5 million/mL cells were placed into each well of six well plates. About 250 ng of the plasmids with the intronic fragments were transfected into the S2 cells with Effectene Transfection Reagent from Qiagen (Cat. No. 301427). Plasmid Blue Script SK (pBS SK) was used as an empty vector in all experiments in order to have equal ng of plasmid in each experiment. For dFOXO experiments, the cells were treated with either 200 ng dFOXO and 50 ng pBS SK or 114 ng pAX (equivalent moles of promoter-containing plasmid) and 136 ng of pBS SK as the control. dFOXO transfections were incubated for 72 h before being assayed. For 20E experiments, the cells were treated with either 250 ng pBS SK and 2 μL of 20-hydroxy ecdysone (10 mg/mL) dissolved in 100% ethanol or 250 ng pBS SK and 2 μL of ethanol as the control. The 20E transfections were incubated for 24 h with the expressed plasmids before adding 2 μL of ethanol or 20E and then incubated for 24 more hours before being assayed at 48 h total.

### Luciferase assays

For luciferase assays, we used the Steady-Luc HTS Assay Kit from Biotium (Cat. No. 30028). Once the transfections were ready to be assayed, the cells were spun down and resuspended in 230 μL Dulbecco’s Phosphate Buffered Saline from Sigma-Aldrich (Cat. No. D8537). The resuspended cells were split into three technical replicates, 65 μL each, and pipetted into 96 well plates, then 65 μL of luciferin substrate was added to each well. Once luciferin was added, the plates were incubated for exactly 10 min before running on a Veritas Luminometer. For each experiment, values were normalized to levels of the parental enhancer’s activity without 20E addition or dFOXO expression.

### Statistical analysis

Luciferase assays for the 300 bp and 600 bp fragments were normalized to the full-size enhancer controls. We used Prism GraphPad Prism9 to perform multiple t-tests analysis of the control vs. treatment to check for statistical significance (GraphPad Prism version 9.0.0 MacOS, GraphPad Software, San Diego, California, USA, www.graphpad.com).

### Sequence conservation analysis

For the sequence alignments shown in Figure 4(b) we used NCBI BLAST using the *Drosophila melanogaster InR* sequence to obtain the *InR* sequences from other species of Drosophila (RefSeq genome sequence GCF_000001215.4) [34]. Sequences were aligned with Clustal Omega to analyse the conservation of the sequences [35]. Using MEME-Suite FIMO, we were able to upload the Drosophila alignment sequences and identify the specific loci of conserved transcription factor binding motifs of interest, here, EcR [36]. For Figure 6, we used the UCSC Genome Browser to analyse 13 Drosophila genomes of varying relatedness to *Drosophila melanogaster* and to compare conservation of regulatory regions [37].

### Site-directed mutagenesis

Primers for site-directed mutagenesis were ordered from IDT and can be found in the primer chart mentioned above (Table 1) [33]. To make small deletions we used the Expand Long Enzyme PCR system from Millipore Sigma (Cat. No.11681834001). After PCR, to digest any methylated DNA and obtain only the mutated plasmid, we digested with the *DpnI* restriction enzyme from New England Biolabs (Cat. No. R0176s).

## Results

### Distinct actions of promoter-proximal enhancers 2, 3, and 4

Our previous studies demonstrated that there are active elements, designated enhancers 2–4, located within 2.3 kbp of the main transcriptional start site that show intrinsic action in Kc and S2 cells and/or are affected by signalling inputs dFOXO and 20E. This segment of regulatory elements is interesting because it appears to integrate signalling inputs in an incoherent fashion, with enhancer 2 stimulated by treatments that reduce enhancer 3 activity. We carried out a fine-structure analysis to identify elements sufficient for regulation, with the goal of understanding possible independent or ‘integrative’ activities [23]. All of the constructs were tested in the context of the T1 promoter, which is the major promoter utilized for *InR* in S2 cells and other cell types. The T1 promoter was previously shown to be the most active transcriptional start site in S2 cells, compared to the T2 and T3 promoters [38]. The T1 promoter region, which extends from −900 to +250, contains more than the core basal promoter and may contribute to regulated expression of the gene. Indeed, although this element has low intrinsic activity in S2 cells, we found that activity of the T1 promoter construct alone was significantly affected by overexpression of dFOXO and 20E treatment (Figures 1(a) and 3(a)). The observed repression effect may be an indirect one, as T1 lacks known binding of EcR from ChIP data, and does not have canonical dFOXO binding motifs.

**Figure 2.**
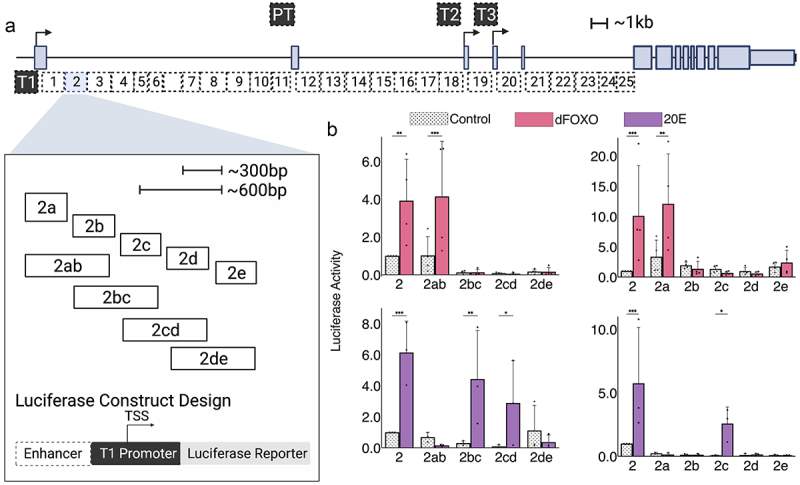
Luciferase reporter analysis of *InR* regulatory region demonstrates that enhancer 2 has separable dFOXO and 20E elements. a) *InR* intronic region divided into 25 ~ 1.5kb fragments (1–25). Enhancer 2 was cloned into smaller 300 bp and 600 bp fragments (2a-2e,2ab-2de). Each enhancer fragment was cloned into a luciferase reporter 5’ of the *InR* T1 basal promoter. b) Enhancer 2 300 bp and 600 bp fragments treated with dFOXO or 20E. In this and following experiments, data represent biological replicates, with at least three experiments per construct. Normalization is relative to activity of the parental enhancer 2. Vehicle controls for 20E treatment were ethanol; for dFOXO, empty expression vector.

**Figure 3.**
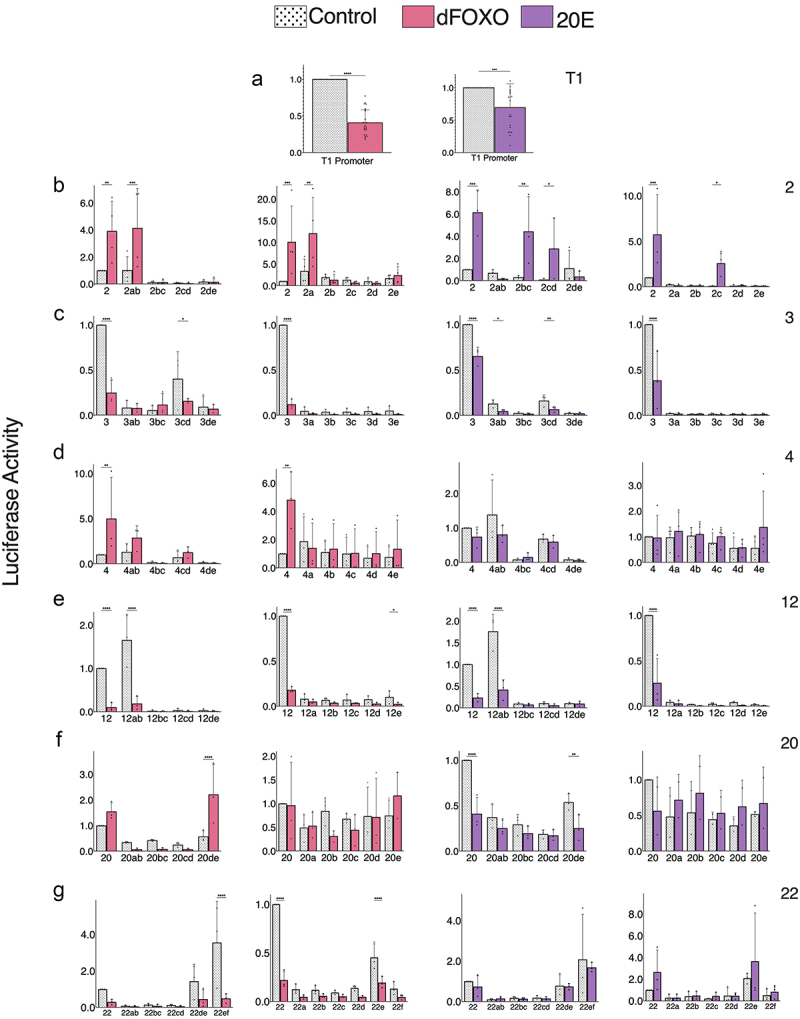
Luciferase reporter analysis of *InR* regulatory enhancers: 300 bp and 600 bp fragments. (a) T1 Promoter response to dFOXO overexpression and treatment with 20E shows inhibitory action of these treatments on the promoter. (b–g) Summary of analysis of enhancers and 300 bp and 600 bp derivatives, including treatment with dFOXO or 20E. Normalized to the parental enhancer control.

Enhancer 2 demonstrates low intrinsic activity in S2 cells but is strongly upregulated upon overexpression with dFOXO or treatment with 20E. We further subdivided this element into 300 bp and 600 bp elements to test for sufficiency of action (Figures 2, and 3(b)). The treatment with dFOXO revealed significant activation centered on the 5’-most 2ab/2a fragment. In contrast, for 20E treatment, the more centrally located 2bc/2 cd/2c fragments were induced by this treatment, while lacking significant activity on their own in the absence of 20E. Our previous serial deletions of the full-length element demonstrated that removal of 2a reduced dFOXO-stimulated activity, and removal of 2c increased the inherent activity of enhancer, consistent with our findings that dFOXO acts through 2a, while 2c has a repression and activation function, that is sufficient and necessary for response to 20E [23].

Enhancer 3 has a high intrinsic activity in S2 cells and is repressed by both dFOXO overexpression and 20E treatment. The 300 bp and 600 bp fragments derived from enhancer 3 showed overall low activity, suggesting that none of these elements are large enough to recapitulate intrinsic activity (Figure 3(c)). Weak intrinsic activity was observed from 3 cd, and possibly 3ab, but the total of these did not equal the intact element; it is likely that multiple binding sites for transcriptional activators distributed throughout the element are needed for function. Our previous serial deletions of enhancer 3 indicated that removal of either 3a, 3c, or 3d severely compromises the output of enhancer 3, thus a ‘distributed’ architecture of activators appears to be characteristic of this element. The negative effects of dFOXO and 20E may reflect indirect effects mediated through the T1 promoter region, which would reduce the potential output of enhancer 3. Alternatively, an indirect genetic cascade may impinge on the enhancer itself. Unlike enhancer 2, it is not clear if these effects are impacting the same or distinct sub-elements of enhancer 3.

Enhancer 4 has low intrinsic activity and is stimulated by dFOXO overexpression but not by 20E treatment. The 300 bp subfragments lacked any dFOXO response, but the 600 bp 4ab fragment appeared to exhibit stimulation by dFOXO (Figure 3(d)). Interestingly, the 4bc and 4de fragments show activity lower than the T1 promoter alone, suggesting the presence of repressors within these elements. The lack of intrinsic activity as well as dFOXO response by any of the 300 bp elements indicates that functionality is distributed over larger elements in enhancer 4. This enhancer was the only dFOXO-inducible element that exhibited cell-type activity (no response in Kc cells) indicating that dFOXO responsiveness may be enabled by distinctive sets of factors present on these enhancers, some of which are restricted to S2 cells.

### EcR long-range repressor activity

**Figure 4.**
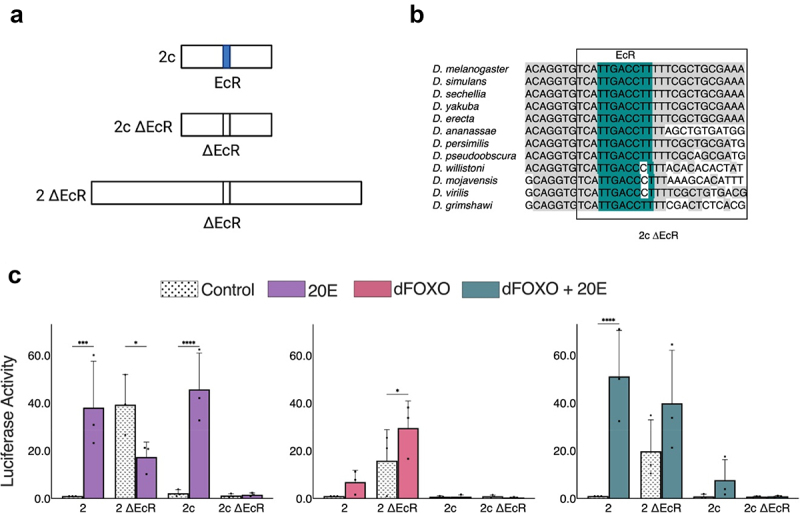
Evidence for long-distance repression by ecdysone receptor of distal dFOXO activated segment of enhancer 2. a) Site-directed mutagenesis to delete EcR binding site localized at enhancer 2c. Deletions were made on the full enhancer 2 and 300 bp fragment 2c. b) Evolutionarily conserved EcR binding site throughout 12 Drosophila species. c) Effects of deletion of EcR binding motif on 2c and full-length enhancer 2 upon treatment with 20E, dFOXO, or dFOXO + 20E. Normalized to the enhancer 2 control.

EcR regulation of *InR* may include direct and indirect effects; previous ChIP experiments have identified binding within region enhancer 2, 4, and 10 (Figure 1(b)). Significantly, one peak overlaps the 2c portion of enhancer 2. We noted that there is an evolutionarily conserved region of this element that includes a highly conserved EcR motif (Figure 4(b)). To test if this EcR binding motif mediated regulation by 20E, we deleted the predicted binding sequence from the 300 bp 2c element (ΔEcR) and compared the activity of the mutant element to the wild-type (Figure 4(a)). The 2c ΔEcR mutant element was inactive and not induced when treated with 20E, indicating that the motif is essential for regulation (Figure 4(c)). Interestingly, we found that the same mutation introduced into the full-length enhancer 2 caused basal activity to significantly increase, to approximately the same level as that of the 20E-stimulated wild-type enhancer (Figure 4(c)). The treatment of this element with 20E reduced activity, perhaps via inhibitory effects on the basal promoter (Figure 3(a)). dFOXO overexpression stimulated both wild-type enhancer 2 as well the enhancer 2 ΔEcR mutant, although the latter had a higher baseline and was further stimulated by dFOXO, indicating that on the wild-type element, EcR repression reduces the potential activity of dFOXO (Figure 4(c)). Treating with both 20E and dFOXO led to net induction of both wild-type and ΔEcR mutant forms of enhancer 2 (Figure 4(c)).

These results suggest that in the wild-type enhancer, repression mediated by the EcR site curtails the activity of distal regulation by activators located within region 2a. The action of EcR over a distance of at least 475 bp is consistent with the activity of long-range repressors, such as Hairy, which have the ability to interfere with distant activators by chromatin modifications that extend over a span of hundreds of bp [39,40]. Our previous serial deletions, taking out large blocks of sequence, are consistent with the distal action of a repressor in 2c and inherent activation by 2a. EcR thus appears to have a bimodal activity; such a repressor-to-activator switch of this factor has been previously observed and is consistent with the conserved mechanisms of other hormone and steroid receptors [41].

### Combinatorial action of enhancers 2 and 3

**Figure 5.**
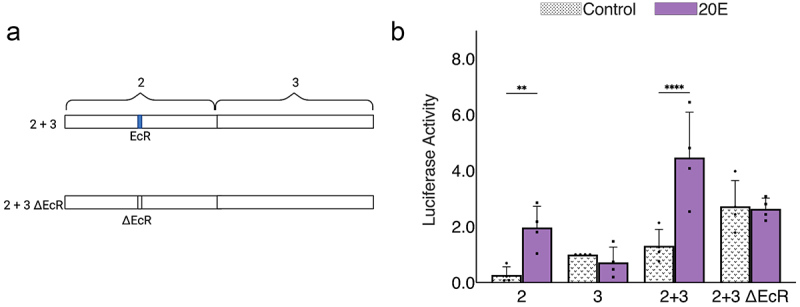
Combinatorial mechanism of enhancer 2 + enhancer 3 with deletion of ecdysone receptor binding site. a) Site-directed mutagenesis to delete EcR binding site on enhancer 2 + enhancer 3. b) Effect of deletion of EcR binding motif on enhancer 2 + enhancer 3 construct upon treatment with 20E. Values were normalized to activity of parental enhancer 3.

The enhancers we defined were arbitrarily divided into 1.5 kbp segments for the sake of analysis, but these sequences are contiguous in the endogenous gene. Regulatory elements located within them may interact or function autonomously to control *InR* promoter activity. Having looked at their independent actions, we combined enhancer 2 and enhancer 3 in their native configuration (Figure 5(a)). Here, we also tested the variant enhancer 2 lacking the EcR motif. The overall activity of wild-type 2 + 3 was similar to 3 alone, which would be consistent with an additive action (Figure 5(b)), while in the presence of 20E, the stimulated activity was greater. There may be elements in these two enhancers that synergize to produce greater than additive outputs in the absence of EcR repression. For the 2 + 3 ΔEcR construct, the activity has a higher baseline activity than wild-type 2 + 3, presumably reflecting the removal of EcR repression action, and here the output appears to be the sum of the potential for enhancer 2 alone with removal of EcR repression (the 20E treatment value) and inherent activity of 3. The 20E treatment of this combination leaves the output unchanged; the somewhat negative effect of 20E on 3 alone appears to be mitigated. Overall, this combination of the 2 + 3 regulatory region appears to produce results that are aligned with an additive model for these enhancers. However, the artificial placement within a reporter plasmid system may bias these results, as enhancer 3 is positioned in a non-physiological promoter proximal position.

### Enhancers 12, 20, and 22

Enhancer 12 possesses a relatively high intrinsic activity, similar to that of enhancer 3. When enhancer 12 is subdivided into the 300 bp or 600 bp fragments, we find that the 3ab element is highly active, even somewhat higher than the parental element. This increase in activity may reflect the loss of a repressive activity in more 3’ portions of the 1.5 kbp element (Figure 3(e)). The closer proximity to the T1 promoter in the luciferase vector may also play a role. Interestingly, none of the 300 bp subelements exhibited much activity, suggesting that dividing a-b in half separates cooperating elements. The response of the active 3ab element to overexpression of dFOXO or treatment with 20E recapitulates the effect seen on the intact 1.5 kbp element, suggesting that transcriptional impacts are mediated by the 3ab portion. The reduction of expression by both of these treatments is much greater than the less-than-twofold effect observed on the T1 promoter alone, suggesting that the enhancer itself mediates this effect. These results are consistent with our previous internal deletions of enhancer 12, which showed that 12a is necessary for activation, and 12c-d mediates some sort of repression [23].

Enhancer 20 is also intrinsically active and is repressed by 20E, while the impact of dFOXO overexpression is ambiguous. In our earlier study, it appeared to be unaffected or repressed, though this effect was not statistically significant. Here we see an apparent induction, but this was similarly not statistically significant. Notably, the 600 bp 20de fragment was significantly upregulated by dFOXO overexpression, but neither the 300 bp 20d nor 20e fragments alone exhibited this effect. The 20E repression was significant for both the parental 1.5 kbp enhancer as well as the 20de fragment. The 300 bp sub-fragments failed to show this effect (Figure 3(f)). Unlike enhancer 2, which has distinct elements for dFOXO and 20E regulation, for enhancer 20, the responses are colocalized on 20de. The previous serial deletions within this 1.5 kbp element showed that 20e, and 20b to a lesser extent, are necessary for intrinsic activity in S2 cells [23]. In these previous studies, removal of 20a or 20b endowed the previously somewhat unresponsive enhancer with a potent induction upon dFOXO overexpression, suggesting that a repressive function within the 5’ portion of enhancer 20 limits dFOXO potential regulation. The 20E regulation, in contrast, was uniformly inhibitory for these mutants, regardless of starting activity. In contrast, in our examination of subfragments, for the 300 bp elements, none were reproducibly regulated by 20E treatment; among 600 bp elements, only 20de (which lacks the inhibitory a-b region) was strongly downregulated by 20E. Overall, these experiments underscore the distinction between dFOXO and 20E regulation on this single enhancer element.

Regulation of enhancer 22 is characterized by activity solely in the 3’ most fragments of both 300 bp and 600 bp, which is suppressed by overexpression of dFOXO, with no significant response to treatment of 20E (Figure 3(g)). Our previous serial deletions of enhancer 22 are consistent with these results, showing the importance of the terminal 600 bp for activity, with a possible repressor activity in the 5’ portion (here, our minimal elements would not reveal a repressor in 22a, since it would be inactive by itself) [23]. Overall, this detailed characterization of the minimal elements sufficient for activity supports our earlier conclusions that dFOXO and 20E have overlapping but separable actions on most enhancers and that the enhancers appear to be integrating contrasting regulatory information, i.e. activation or repression by these signals. The detailed dissection of these elements also reveals the long-range effect of EcR repression within the enhancer 2 region, which does not appear to extend more broadly across the enhancer 2–3 region.

### Evolutionary conservation

**Figure 6.**
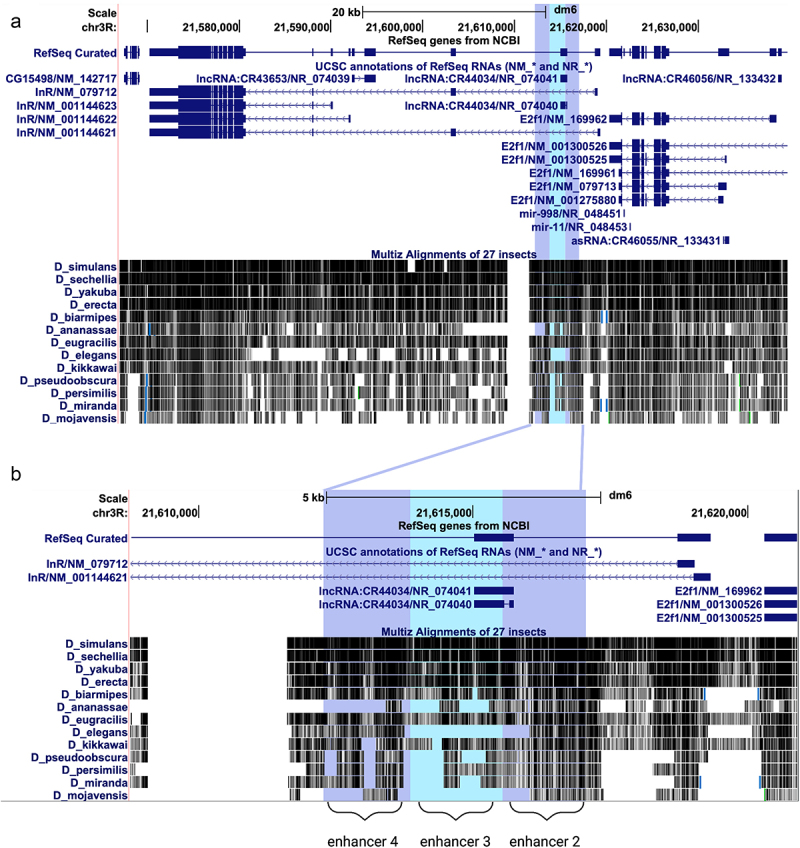
Evolutionary conservation of the *InR* locus. a) Conservation of the entire *InR* locus in 13 Drosophila species from the UCSC Genome Browser. b) Zoomed in view of the integrated locus of enhancers 2, 3, and 4, illustrating the higher level of conservation in enhancer 2.

Having defined the functional elements of *InR* necessary and sufficient for activity in S2 cells, it is interesting to speculate on the conservation of such cis-regulatory circuitry. Within Drosophila, the protein coding exons of *InR* are highly conserved throughout evolution, as expected, while the intronic regions are less conserved (Figure 6(a)). Enhancer sequences are difficult to identify with sequence analysis alone, although these sequences are evolutionarily constrained and can be enriched in functional variants relevant to complex traits and disease [42]. However, by combining information from reporter assays, evolutionary conservation, and chromatin marks, we can obtain a clearer picture of *InR* regulatory elements. Considering the cluster of promoter-proximal enhancers (2–4), enhancer 2 is more highly conserved than enhancers 3 and 4, perhaps underscoring the importance of the direct EcR regulatory switch in regulating *InR* (Figure 6(b)).

## Discussion

EcR has been shown to regulate hundreds of loci in Drosophila, although many genes controlled by 20E represent secondary or tertiary effects of the hormone. Direct regulation by EcR has been demonstrated in a number of cases. For instance, EcR binds to promoter-proximal regions of *hsp27* and *fbp1* and greatly stimulates their expression upon exposure to 20E [43]. This on/off effect represents an important mechanism for driving cell- and developmental-specific gene expression. Notably, about one half of identified EcR interaction sites identified in Kc cells did not result in changes to gene expression of nearby genes, but many of these ‘unresponsive’ sites showed evidence of 20E regulation in other cell types [29]. Therefore, EcR may require additional factors in some instances to enable hormonal regulation. We see evidence of this phenomenon on *InR* as well. ChIP-seq identified EcR binding sites within the insulin-like receptor gene in S2 cells on enhancers 2, 4, and 10 (Figure 1(b)). The only enhancer to have an ecdysone response in S2 cells, however, was enhancer 2. Enhancer 4 exhibited no significant response to ecdysone, and enhancer 10 had a response only in Kc cells [23]. This highlights the intricacy of EcR regulation, and that the other EcR binding sites in enhancer 4 and 10 may represent enhancers only active in a cell-type specific manner. Thus, further defining the functional significance of EcR binding requires direct experimentation.

By making use of transfected reporters in Drosophila cell culture, we uncovered novel aspects of *InR* regulation, in particular, the direct regulation by EcR and the range of repression mediated by this factor when not liganded to 20E. Previous studies demonstrated that EcR is found not only at the promoter but also in distal locations, such as those on early genes such as *E75, E74*, and *Broad* [44]. On these genes, stimulation by 20E induces the formation of enhancer-promoter loops that reflect promoter activation. These genes are, however, not as widely expressed as *InR* is, and these studies did not explore whether EcR was functioning as an active repressor in the absence of hormone, although previous studies have found that EcR can indeed associate with corepressors [45,46]. Thus, the question arises whether EcR mediates a binary on/off activity on *InR* as well. Our findings indicate that within the *InR* intronic region comprising enhancer 2, EcR locally represses activators found within the 2a segment of the enhancer. In Drosophila, transcriptional repressors have been characterized as ‘short-range’ or ‘long-range’ based on their ability to act on neighboring activators. Short-range repressors like Snail and Giant exert inhibitory effects only when bound within 100 bp of activators, while in contrast, long-range repressors such as Hairy can inhibit activators up to several kilobase pairs of the targeted activators. These distinct regulatory effects are correlated with correspondingly local or broad chromatin modification [40,47–49]. EcR repression from segment 2c to segment 2a (~500–800 bp), is consistent with the long-range mechanism. The repressive activity appears to have a limit, such that enhancer 2 repression does not appear to affect the activity of enhancer 3 when the enhancers are combined (Figure 5(b)). Thus, a locally-acting binary operation by EcR, embedded in a larger field of regulatory elements, may provide *InR* with a steadier output, suitable for this broadly expressed gene. Such localized repression function may also explain how repressors and corepressors participate in so-called ‘soft repression’ [50].

FOXO regulation of *InR* has been previously identified as an important feedback mechanism that may potentiate insulin signalling in Drosophila as well as mammals [8]. Previous studies in Drosophila have suggested that dFOXO may interact with the gene through internal promoters; however, the experimental evidence for this is weak [51] There is limited information regarding *in vivo* binding by this transcription factor in Drosophila. A study exploring dFOXO binding in the adult female identified ~700 transcriptional targets, with dFOXO bound to the coding region of *InR* in adult females [52]. The protein was also previously shown to bind the T1 promoter in S2 cells [7,8]. Our previous ChIP identified dFOXO binding activity within the *InR* gene in S2 cells. Yet, the most prominent peaks on region 10 did not correspond to dFOXO responsiveness, and binding at enhancers 2 and 4, which are stimulated by dFOXO overexpression, was barely above background [23]. Thus, predicting dFOXO responses from ChIP binding data is challenging. Similarly, we found that the presence of dFOXO-like binding motifs does not appear to correlate with dFOXO response in S2 cells (data not shown). The insulin signalling pathway triggers a well-established process of FOXO phosphorylation and nuclear exclusion, a process that is conserved from insects to mammals. However, FOXO regulation involves additional signalling pathways, including MST1 in *C. elegans* and JNK in mice, pointing to further complexities of this regulatory system [53,54]. It is possible that our transient transfection assays, with dFOXO overexpression, may distort the true response of the enhancers to dFOXO; subsequent mutation of individual dFOXO-responsive elements within the endogenous gene, such as in 2a and 4ab, will be illuminating. Our studies did not conclusively determine whether the inhibitory effect of dFOXO on the T1 promoter and other enhancers is direct or indirect, but given the 72-h timeline for the procedure, there would be time enough for known growth-inhibitory effects of dFOXO to broadly interfere with gene expression.

The use of reporter genes is a widespread and powerful tool for detailed assessment of regulatory regions. However, there are clear limitations to this reductionist approach when trying to gain an understanding of a large and complex regulatory locus. First, the demarcations of individual enhancers are arbitrary, carried out for efficiency in designing transfection assays. We show that many of the defined enhancers lose activity when divided into smaller fragments, thus there is every reason to expect that some of the divisions between the original 1.5 kbp elements fatally subdivided a more complex element. We did, however, find that some elements, notably enhancer 2, were readily subdivided to identify the dFOXO and EcR responsive portions. Furthermore, we were able to test the function of larger regions; by combining enhancer 2 and enhancer 3, we discovered that these enhancers appear to work additively, but with treatment of 20E, it appears that the combined region may be superstimulated, suggesting a synergistic action (Figure 5(b)). On the endogenous locus, we imagine that the enhancers may work additively, or they may antagonize or synergize. We are intrigued by how a site-directed mutagenesis on the endogenous locus to delete the enhancer 2 EcR binding site may impact the expression of *InR*. From our current understanding of this complex regulation, we believe that there would be an overexpression of *InR* during fasting due to the dFOXO activator found at enhancer 2a, and lower expression would be seen during development when ecdysone levels are high. Clearly, to establish this sort of function, we will need to examine the function of the mostly intact, endogenous locus – a task that has become much simpler in an era of CRISPR tools. However, in sum, the use of transfected reporters has allowed us to move considerably beyond our initial assessment, from defining elements that are necessary to elements that are sufficient for certain responses. Thus, this reductionist analysis of each enhancer can help us better understand the regulation of *InR*. Future work will focus on the endogenous locus, using diverse techniques to understand how these enhancers work as an ensemble. Delving into the mechanisms by which this gene is regulated will help us understand natural variation, providing insights into pathological states, development, and evolution.
